# Secular changes in eruption of primary teeth in Chinese infants and young children from three national cross-sectional surveys

**DOI:** 10.1038/s41598-024-59044-0

**Published:** 2024-04-08

**Authors:** Ya-qin Zhang, Xin-nan Zong, Hua-hong Wu, Hui Li

**Affiliations:** Department of Growth and Development, Capital Institute of Pediatrics, Beijing, 100020 China

**Keywords:** Infants, Primary teeth, Eruption age, Secular changes, Developmental biology, Health care, Medical research

## Abstract

The eruption of primary teeth is a basic event during physical development of children, which is affected by heredity and environment. This study aimed to analyze the changes in primary teeth eruption among Chinese children with social development. A total of 249,264 healthy children under 2 years were extracted from the 1995, 2005, and 2015 National Survey on the Physical Growth and Development of Children in Nine Cities of China. Their primary teeth were examined and percentiles of primary teeth eruption age were calculated by probit analysis. The median primary teeth eruption age were 6.8 months, 6.7 months, 6.6 months in 1995, 2005 and 2015. Primary teeth eruption age of boys was 0.2 months, 0.3 months, 0.3 months earlier than that of girls in 1995, 2005 and 2015. Primary teeth eruption age was the earliest in children from northern region and was the latest in children from southern region, and this regional difference did not change over time. These findings suggest that primary teeth eruption age slightly advanced with social development, and their gender difference and regional difference have always existed, which supplied some data for understanding the secular trend of primary teeth development in stomatology, pediatrics, anthropology, and other related fields.

## Introduction

Teeth development in children is considered to be a primary marker of human physiological maturity in addition to skeletal and pubertal sexual development^1^. As an important milestone in the growth and development of infants and young children, primary teeth development may supply some important information for evaluation of children's development, biological age determination, and early identification of potential growth and development related diseases.

Teeth development is regulated by both heredity and environment^2^, which varies among populations^3^. Meanwhile, with changes in the social environment, secular changes in children's growth and development have observed, for example, the height increased and the age at menarche and thelarche decreased^4,5^. Similarly, whether there is also a secular change in teeth development and how it changes is also a matter of concern. It not only provides basic data for anthropology research, but also helps to understand and timely update the reference data for evaluation of teeth development. At the same time, it can provide some reference evidence for formulating policies in the oral health care and the early prevention of dental caries. Some studies on permanent teeth development show that the eruption age of permanent teeth displays an advancing trend in varying degrees^6–9^. However, studies on secular changes in primary teeth development are relatively rare, and the existing reports are not completely consistent and sometimes even contradictory^10–16^. Therefore, it is still necessary to conduct extensive analysis on primary teeth eruption in more populations in order to further reveal the secular trend of primary tooth development.

“The National Survey on the Physical Growth and Development of Children in Nine Cities of China” (NSPGDC) is a large-scale cross-sectional survey of the growth and development of children, which has been conducted five times by 2015^17^. NSPGDC began the collection of primary teeth development data with the same methodology in 1995, 2005 and 2015. These series studies amassed valuable data for exploring the secular trend of infant primary tooth development. This population bases study aimed to investigate whether there was a change in primary teeth development during the two decades in China, which may supply some data for the understanding of the secular change in primary tooth development of global populations and for the future research in stomatology, pediatrics, anthropology, and other related fields.

## Methods

### Data source

This study was a secondary analysis of data derived from the NSPGDC III–V. The NSPGDC III–V were cross-sectional surveys based on large-scale healthy populations and conducted across nine cities during June-October in 1995, 2005 and 2015, respectively. The nine cities included Beijing (Municipality), Harbin (Heilongjiang’s provincial capital), Xi’an (Shannxi’s provincial capital), Shanghai (Municipality), Nanjing (Jiangsu’s provincial capital), Wuhan (Hubei’s provincial capital), Guangzhou (Guangdong’s provincial capital), Fuzhou (Fujian’s provincial capital), and Kunming (Yunnan’s provincial capital). Beijing, Harbin, Xi’an were classified as northern region, Shanghai, Nanjing, Wuhan as central region, and Guangzhou, Fuzhou, Kunming as southern region according to their latitudes^17^. All of these surveyed sites were nearly the same in the NSPGDC III–V. Multistage stratified cluster sampling method was used according to administrative districts in each city. Communities in each selected administrative district were considered as the cluster sample unit.

### Participants and sample size

Healthy children under 2 years old who are local residences in the selected region or those who lived in the region for longer than 2/3 of their lifetimes were evaluated for the status of their primary teeth development in the three surveys. The inclusion and exclusion criteria of participants in the three surveys were the same, as reported previously^17^. All children were divided into 12 age groups: 1 m ~ , 2 m ~ , 3 m ~ , 4 m ~ , 5 m ~ , 6 m ~ , 8 m ~ , 10 m ~ , 12 m ~ , 15 m ~ , 18 m ~ , and 21 ~ 24 m. The 1 m ~ group included children whose ages ranged from 1 complete month to one day less than 2 months and other groups were analogously defined. The total sample size was 249,264 and 85,454 in 1995, 74,935 in 2005 and 88,875 in 2015. Table 1 shows sex-age subgroup sample sizes.

**Table 1:** Sample size of the NSPGDC III–V (1995–2015).

Age group	1995			2005			2015		
	Boys	Girls	Total	Boys	Girls	Total	Boys	Girls	Total
1 m ~	3537	3487	7024	3127	3109	6236	3715	3703	7418
2 m ~	3549	3510	7059	3067	3057	6124	3664	3605	7269
3 m ~	3543	3522	7065	3104	3109	6213	3719	3731	7450
4 m ~	3548	3509	7057	3137	3132	6269	3613	3589	7202
5 m ~	3562	3509	7071	3104	3094	6198	3601	3608	7209
6 m ~	3600	3562	7162	3165	3152	6317	3770	3733	7503
8 m ~	3600	3544	7144	3155	3160	6315	3758	3747	7505
10 m ~	3577	3546	7123	3129	3100	6229	3729	3751	7480
12 m ~	3599	3550	7149	3150	3133	6283	3777	3742	7519
15 m ~	3600	3600	7200	3166	3132	6298	3715	3731	7446
18 m ~	3600	3600	7200	3143	3099	6242	3765	3750	7515
21 ~ 24 m	3600	3600	7200	3113	3098	6211	3724	3635	7359
Total	42,915	42,539	85,454	37,560	37,375	74,935	44,550	44,325	88,875

### Ethics approval and consent to participate

The study had been approved by the Ethics Committee of the Capital Institute of Pediatrics (SHERLL2015009). All participations were voluntary and the inform consents were obtained from the parents of all participants after members of the surveys’ staff explained to the parents of children the purpose of the survey. Additionally, all methods were performed in accordance with the Declaration of Helsinki.

### Data collection and quality control

Primary teeth development was examined in the field by trained investigators using the same method in the three surveys. Primary teeth eruption was defined as having occurred if any part of the primary teeth had, on direct inspection of the mouth, pierced the gum line (clinical emergence). The status of primary teeth eruption was recorded as yes or no in the customized questionnaire and the total number of erupted primary teeth was also recorded. Furthermore, weight, length and head circumference were measured by investigators using the standardized method, and other related information (paternal demographic data, breastfeeding, complementary feeding) was obtained by interview in person. All investigators participated in rigorous specialized training prior to collection of data and passed an examination before the survey. 5% of total subjects in each site were measured repeatedly at random each day, and the proportion of subjects beyond allowable errors was less than 10%.

### Statistical analysis

Data analysis was done using the Statistical Package for the Social Sciences (SPSS) software, version 22 (developed by SPSS Inc. in Chicago, IL, USA). Z scores of weight, length, head circumference and BMI were calculated by LMS method using the WHO standards (2006). All the basic characteristics were expressed as frequencies for categorical data and‾x ± SD for quantitative data, and their corresponding comparison among the three NSPGDC (III–V) were analyzed by χ^2^ or Analysis of Variance (ANOVA) method. Comparison of the primary teeth eruption prevalence in each age group among the NSPGDC III–V was carried out by linear-by-linear association χ^2^ method. The probit regression analysis, the conventional way to determine the age of transition into any developmental stage which was used to determine the cumulative percentage of subjects by month or year of age entering into the developmental stage as has been previously described^18^, was performed to calculate the percentiles (the 3rd, 10th, 25th, 50th, 75th, 90th and 97th) of eruption age of primary teeth. The median and the quartiles values (Q1,Q3) of the number of erupted primary teeth were calculated and their comparison among the three surveys was analyzed by the independent sample Kruskal–Wallis Test. The eruption age of the primary teeth and the number of erupted primary teeth were compared with descriptive methods in 1995, 2005 and 2015, which detailed the secular changes of primary teeth development for Chinese infant and young children. A value of *P* < 0.05 was considered statistically significant.

## Results

### Basic characteristics of participants in the NSPGDC III–V, 1995–2015

The basic characteristics of participants in 1995, 2005 and 2015 is shown in Table 2. We found there was no statistical difference in the proportions of gender or regions in the three surveys. The level of parental education significantly improved during the two decades and the family income also increased during the lasted decade. Birth weight of participants slightly increased from 1995 to 2015. In addition, the length and weight of children significantly increased during the two decades. The BMI and head circumference increased significantly in the first decade and their changes were not obvious in the second decade.

**Table 2:** Basic characteristics of participants in the NSPGDC III–V, 1995–2015.

	1995	2005	2015	*P value*^*a*^
Gender, n (%)				
Male	42,915 (50.2)	37,560 (50.1)	44,550 (50.1)	0.904
Female	42,539 (49.8)	37,375 (49.9)	44,325 (49.9)	
Region, n (%)				
Northern	28,759 (33.7)	25,191 (33.6)	30,108 (33.9)	0.706
Central	28,686 (33.6)	25,248 (33.7)	29,873 (33.6)	
Southern	28,009 (32.8)	24,496 (32.7)	28,894 (32.5)	
Father education, n (%)				
College degree and above	13,030 (15.2)	21,113 (28.2)	46,901 (52.8)	< 0.001
Senior school/ technical secondary school	26,180 (30.6)	24,911 (33.2)	22,850 (25.7)	
Junior middle school	40,587 (47.5)	26,420 (35.3)	17,691 (19.9)	
Primary school and below	5594 (6.5)	2460 (3.3)	1327 (1.5)	
Unknown	63 (0.1)	31 (0.0)	106 (0.1)	
Mother education, n (%)				
College degree and above	9439 (11.0)	17,978 (24.0)	45,758 (51.5)	< 0.001
Senior school/technical secondary school	25,491 (29.8)	23,962 (32.0)	22,627 (25.5)	
Junior middle school	42,704 (50.0)	29,223 (39.0)	18,836 (21.2)	
Primary school and below	7751 (9.1)	3743 (5.0)	1538 (1.7)	
Unknown	69 (0.1)	29 (0.0)	116 (0.1)	
Family income, n (%)				
≤ ￥30,000	–	52,061 (69.5)	6742 (7.6)	< 0.001
￥30,000 ~ 49,999	–	10,292 (13.7)	21,505 (24.2)	
￥50,000 ~ 99,999	–	6596 (8.8)	33,036 (37.2)	
￥100,000 ~ 299,999	–	1139 (1.5)	23,504 (26.4)	
≥ ￥300,000	–	107 (0.1)	3682 (4.1)	
Unknown	–	4740 (6.3)	406 (0.5)	
BW(g)^b,c^	3300 (401)	3347 (414)	3357 (440)	< 0.001
LAZ^b,d^	− 0.06 (1.11)	0.31 (1.09)	0.37 (1.03)	< 0.001
WAZ^b,e^	0.13 (0.93)	0.37 (0.97)	0.41 (0.95)	< 0.001
BMIZ^b,f^	0.24 (0.95)	0.27 (0.99)	0.27 (0.98)	< 0.001
HCZ^b,g^	− 0.02 (0.96)	0.15 (1.01)	0.02 (0.98)	< 0.001

^a^*P values* were determined using analysis of variance (ANOVA) test for continuous variables and Chi-square test for categorical variables.

^b^Data are given as means, with standard error (SE) in parentheses.

^c^Birth weight.

^d^Length for age Z-score.

^e^Weight for age Z-score.

^f^Body mass index for age Z-score.

^g^Head circumference for age Z-score.

### Changes in prevalence of primary teeth eruption at the population level

Table 3 shows that the prevalence of primary teeth eruption in the age group of 5 ~ 10 months groups increased from 1995 to 2015 (*P* = 0.000 ~ 0.036), while its changes in other age groups from 1995 to 2015 were no statistical significance.

**Table 3:** Changes of prevalence of primary teeth eruption in different age groups. n refer to the number of children whose primary teeth had erupted, % refer to the prevalence of primary teeth eruption in the age group.

Age group	1995		2005		2015		*P value*
	n	%	n	%	n	%	
1 m ~	0	0.0	0	0.0	0	0.0	–
2 m ~	0	0.0	0	0.0	0	0.0	–
3 m ~	12	0.2	12	0.2	21	0.3	0.145
4 m ~	182	2.6	158	2.5	197	2.7	0.557
5 m ~	834	11.8	753	12.1	979	13.6	0.001
6 m ~	2932	40.9	2662	42.1	3227	43.0	0.011
8 m ~	5752	80.5	5081	80.5	6144	81.9	0.036
10 m ~	6776	95.1	5990	96.2	7204	96.3	0.000
12 m ~	7109	99.4	6246	99.4	7478	99.5	0.905
15 m ~	7197	100.0	6298	100.0	7441	99.9	–
18 m ~	7200	100.0	6241	100.0	7514	100.0	–
21 ~ < 24 m	7200	100.0	6211	100.0	7355	99.9	–

### Changes in eruption age of primary teeth

Table 4 shows the median age of primary teeth eruption was 6.8 months in 1995, 6.7 months in 2005, and 6.6 months in 2015, which declined 0.2 months during the two decades. The relative change of the median age of primary teeth eruption was 2.9% (calculated by “the changes during 1995–2015 divided by the value in 1995, 0.2 months/6.8 months * 100”). Furthermore, the decrement of the 3rd and 97th percentile age of primary teeth eruption was respectively 0.1 months and 0.3 months during the two decades.

**Table 4:** Changes of the percentiles of primary teeth eruption age. ^a^ the eruption age in 2015 minus that in 1995.

Percentile	1995		2005		2015		Differences of Eruption age,1995–2015	
	Eruption age	95%CI	Eruption age	95%CI	Eruption age	95%CI	Difference^a^	95%CI
3rd	**4.2**	4.15 ~ 4.24	**4.2**	4.12 ~ 4.20	**4.1**	4.04 ~ 4.12	**−** **0.1**	**−** **0.13 ~ −** **0.07**
10th	4.9	4.85 ~ 4.93	4.8	4.80 ~ 4.89	4.8	4.72 ~ 4.80	**−** 0.1	**−** 0.12 ~ **−** 0.08
25th	5.7	5.66 ~ 5.75	5.7	5.61 ~ 5.70	5.6	5.51 ~ 5.59	**−** 0.2	**−** 0.14 ~ **−** 0.06
**50th**	**6.8**	**6.73 ~ 6.82**	**6.7**	**6.67 ~ 6.77**	**6.6**	**6.55 ~ 6.64**	**−** **0.2**	**−** **0.23 ~ −** **0.17**
75th	8.0	7.99 ~ 8.1	8.0	7.91 ~ 8.04	7.8	7.77 ~ 7.89	**−** 0.2	**−** 0.30 ~ **−** 0.10
90th	9.4	9.31 ~ 9.47	9.3	9.23 ~ 9.39	9.1	9.06 ~ 9.21	**−** 0.3	**−** 0.39 ~ **−** 0.21
97th	**10.9**	10.83 ~ 11.04	**10.8**	10.74 ~ 10.95	**10.6**	10.55 ~ 10.75	**−** **0.3**	**−** **0.45 ~ −** **0.15**

**Figure 1:**
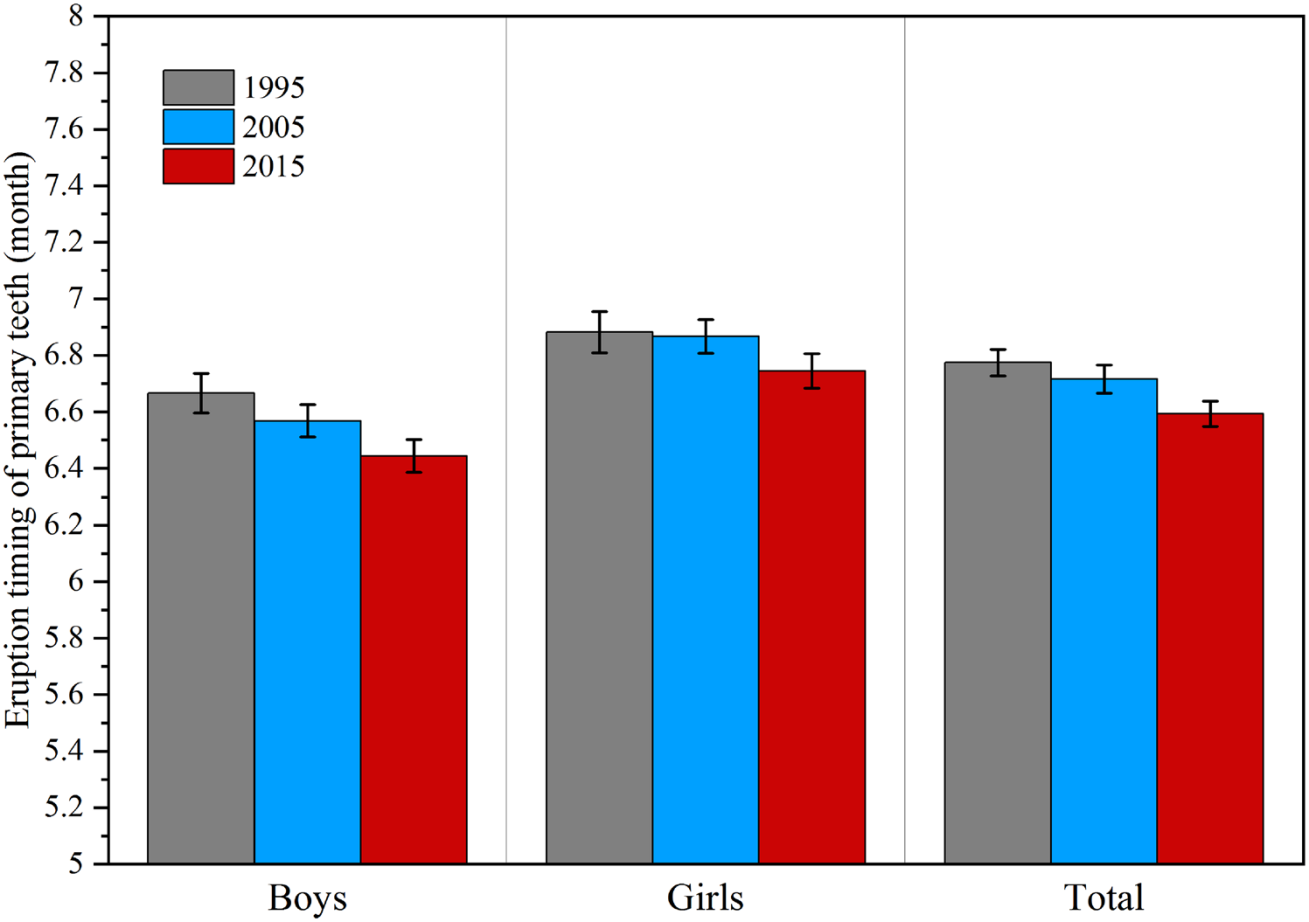
The median eruption age of primary teeth in boys and girls from 1995 to 2015. The error bar was the 95%CI of the median eruption age of primary teeth.

Figure 1 shows that the median age of primary teeth eruption of boys were 6.7 months in 1995, 6.6 months in 2005 and 6.4 months in 2015, and those of girls were 6.9 months in 1995, 6.9 months in 2005 and 6.7 months in 2015. Throughout the three surveys, the primary teeth eruption age of boys were earlier than that of girls, and the gender difference was always remain, such as 0.2 months in 1995 and 0.3 months in 2015.

**Figure 2:**
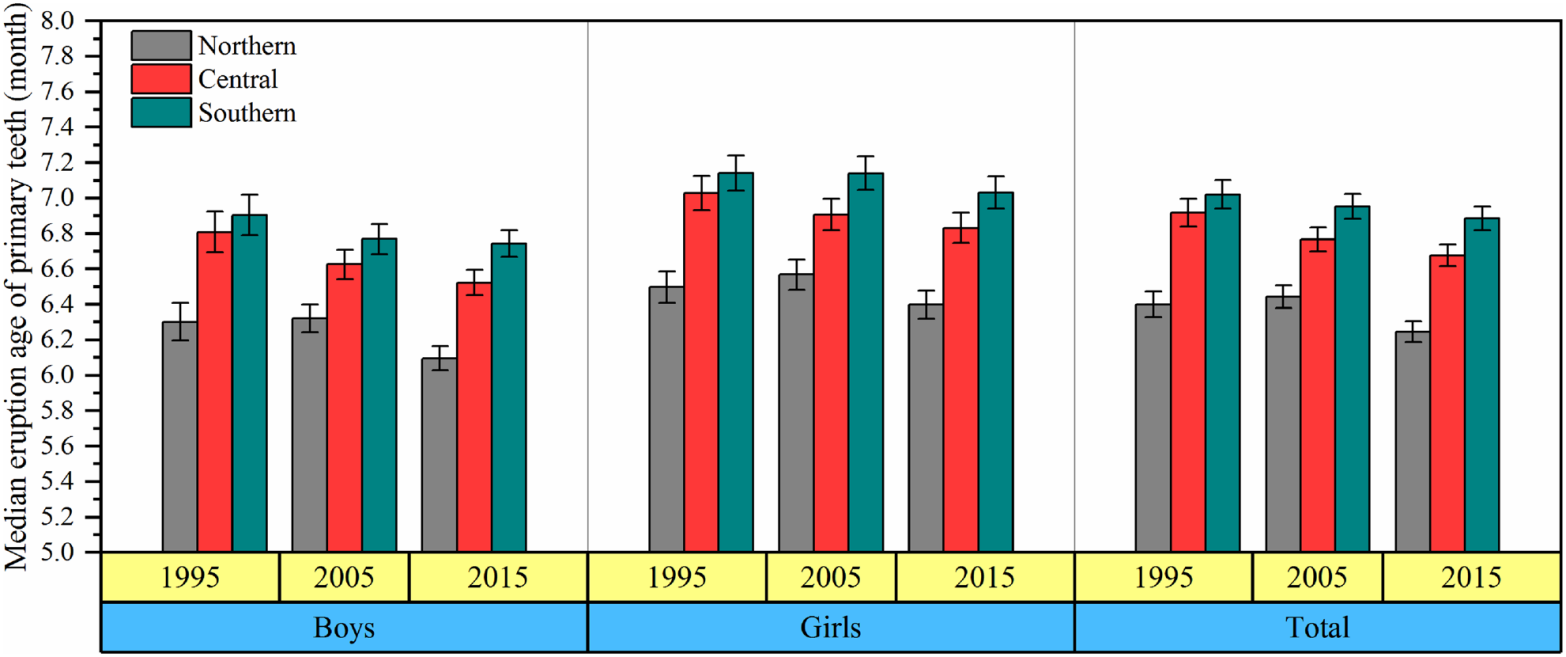
The median eruption age of primary teeth in northern, central and southern regions from 1995 to 2015. The error bar was the 95%CI of the median eruption age of primary teeth.

Figure 2 shows that the eruption age of primary teeth was the earliest in children from the northern region and the latest in the southern region in the three surveys, and the difference between the northern and southern region was 0.5–0.6 months from 1995 to 2015.

### Changes in number of erupted primary teeth in the same age group from 1995 to 2015

**Figure 3:**
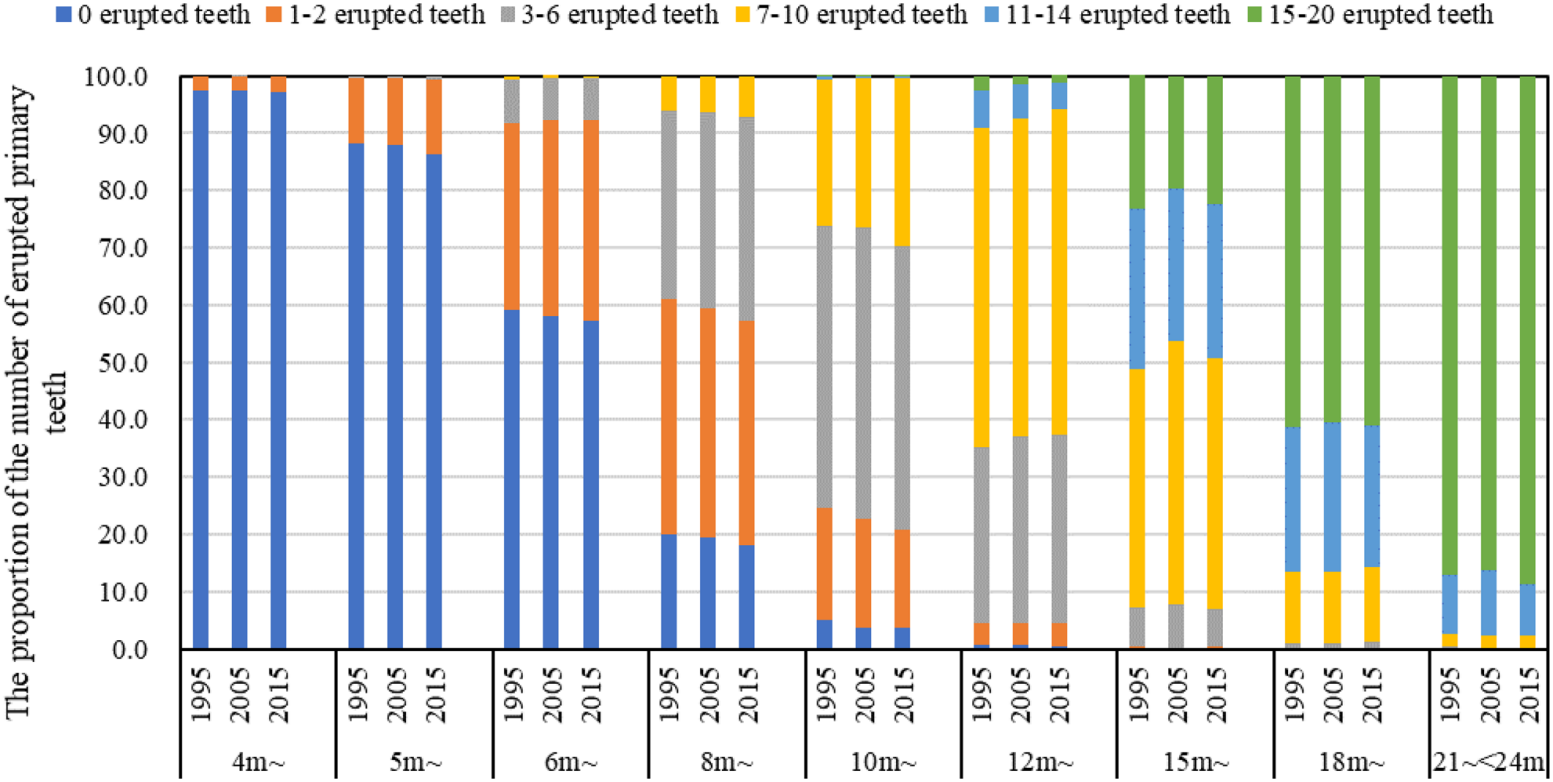
The proportion of the number of erupted primary teeth in each age group in 1995, 2005 and 2015.

Table 5 shows the number of erupted primary teeth in each age group in 1995, 2005 and 2015, whose difference were statistical significance at 7 m ~ group to 15 m ~ group during 1995–2015. For example, the median of the number of erupted primary teeth at 10 m ~ group was 4 in 1995, 5 in 2005 and 5 in 2015. Figure 3 shows the proportion of the number of erupted primary teeth in each age group in the three surveys, which illustrated that the proportion of infants with more number of erupted primary teeth in the same group was higher in 2015 than that in 1995, for example, the proportion of 0 erupted teeth, 1–2 erupted teeth, 3–6 erupted teeth and ≥ 6 erupted teeth was respectively 20.0%, 40.9%, 33.1% and 6.0% in 1995, and 18.1%, 39.1%, 35.6% and 7.1% in 2015 (*P* = 0.000).

**Table 5:** Number of erupted primary teeth in each age group in 1995, 2005 and 2015.

Age group	1995			2005			2015			*P value *^*a*^
	N	Median	Q1, Q3	N	Median	Q1, Q3	N	Median	Q1, Q3	
1 m ~	7024	0	0–0	6236	0	0–0	7418	0	0–0	–
2 m ~	7059	0	0–0	6124	0	0–0	7269	0	0–0	–
3 m ~	7065	0	0–0	6213	0	0–0	7450	0	0–0	–
4 m ~	7057	0	0–0	6269	0	0–0	7202	0	0–0	0.700
5 m ~	7071	0	0–0	6197	0	0–0	7208	0	0–0	0.003
6 m ~	7162	0	0–2	6315	0	0–2	7495	0	0–2	0.121
8 m ~	6948	2	1–4	6314	2	1–4	7501	2	2–4	0.000
10 m ~	7123	4	3–7	6228	5	3–7	7477	5	3–7	0.000
12 m ~	7149	8	6–8	6283	8	6–8	7515	8	6–8	0.000
15 m ~	7200	11	8–14	6298	10	8–14	7446	10	8–14	0.000
18 m ~	7200	16	12–16	6242	16	12–16	7515	16	12–16	0.080
21 ~ < 24 m	7200	16	16–17	6210	16	16–16	7352	16	16–16	0.000

N was sample size, Median was the median of the number of erupted primary teeth, Q1 was the 25th percentile of the number of the erupted primary teeth, Q3 was the 75th percentile of the number of erupted primary teeth. ^a^*P values* were determined using analysis of Kruskal Wallis test among 1995, 2005 and 2015.

## Discussions

Under the background of the secular trend of physical growth in children, it is also critical to understand whether there is a short-term or long-term change in the primary teeth eruption for the evaluation of tooth development, the formulation of early prevention policies for dental caries and other anthropological studies. In the present study, we observed a change in primary teeth eruption from 1995 to 2015 in Chinese infants and young children.

The prevalence of primary teeth eruption from 1995 to 2015 increased slightly in infants aged 5 ~ 10 months, but it did not significantly change in infants under 5 months and older than 12 months. These results suggested that early or delayed eruption of primary teeth was not common in infants in the two decades, but more infants aged 5 ~ 10 months have erupted primary tooth today than those of 20 years ago. Furthermore, we found that the median eruption age of primary teeth has declined from 1995 to 2015. A similar secular trend was observed in Polish children^14^ and in Caucasian infants from Arizona^11^. However, other reports showed a delayed trend in Indian^15^ and Egyptian infants^13^. Whether the differences in the secular trends of primary teeth development among various populations are related to ethnic and social development factors remains unclear due to lack of evidence. But it is worth noting that we found that data on the secular changes of primary teeth eruption usually came from some different surveys which were various in study design, selection of subjects, data collection and analysis methods by analyzing the existing reports. These differences may have an impact on the data comparability and one should exercise caution when explaining secular trends of primary teeth development using these data^12^. It is suggested that scientific evaluation of more primary teeth development data from various populations based on the same research methods is necessary to clarify whether there is an advanced trend on primary teeth development and its ethnic differences in the future study.

Percentiles were used to show the distribution of primary teeth eruption age. We found that the changes in higher percentiles of primary teeth eruption age were slightly greater than that in the middle and lower percentiles. These results indicate that the range of primary teeth eruption age tends to narrow slightly. Some studies have shown that the delayed eruption of primary teeth is closely related to nutritional status^19^, lower socioeconomic status, secondary maternal education and lower birth weight^20,21^, which suggests that the phenomenon of delayed or late eruption of primary teeth may be improved with nutrition improvement and social development. Our baseline data shows that parental education level, family income, birth weight and nutritional status (weight, length and BMI) of infants have significantly improved over the two decades, which may be related to the advance of the age of late primary teeth eruption and the narrowing of the range of eruption age. It is suggested that the primary teeth eruption will advance with social development, economic improvement and nutritional improvement during pregnancy and infancy, especially the situation of delayed teeth eruption will be significantly improved.

Furthermore, we found that the relative changes of the median age of primary teeth eruption were 2.9%. However, a systematic review showed that the age of breast development in Chinese girls advanced with a relative change of 6.1% (0.6 years/9.8 years * 100) over 17 years^5^, and the age of menarche in Chinese girls advanced with a relative change of 7.0% (0.94 years old/13.41 years old * 100) over 25 years^22^. It can thus be seen that the relative change of eruption age of primary teeth was lesser than other physical development indicators, which suggest that the effect of social environment on primary teeth development may be weaker and then its secular changes showed a smaller trend.

Surveys in Canada^23^, the United States^11^, Spain^24^ and Poland^14^ have shown that the eruption age of primary teeth in boys is slightly earlier than that in girls, and this gender difference is related to the different hormone levels between genders during fetal dentition development^25^. Similar results were also found in our study, and the changes in gender difference of primary teeth eruption age were not obvious. The results suggested that the gender difference of primary teeth development may be mainly related to physiological differences between genders and may not change significantly with environment.

There are obvious regional differences in physical growth of Chinese children^17^. In the present study, we also found that the regional difference in the primary teeth eruption age, which illustrated that the primary teeth eruption age in the northern region is earlier than that in the southern region, and this regional difference does not narrow with time. Other data from China also indicated the similar regional difference, for example, the eruption age of primary teeth in Gansu province (7 months) in northern China^26^ was earlier than that in Hong Kong (8.2 months)^27^ and Chongqing (8 months) in the south^28^. The regional differences in primary teeth development may be related to the great differences in climate, living environments, cultural customs, eating habits, parental somatotypes in different regions.

In addition to the eruption age, the number of erupted primary teeth are also important. We found that the proportion of infants with more number of erupted primary teeth in the same group became higher over the two decades, which suggested that the number of erupted primary teeth in the same age group increased. A recent report in Japan shows that the mean number of emerged primary teeth decreased between 1980 and 2012, and believed that this trend associated to the decrease of birth weight and weight and height at 18 months old^16^. The difference in changes of the number of emerged primary teeth may relate to the increment of birth weight, length and weight of infants in our study. It was indicated that the number of erupted primary teeth will increase with nutritional improvement during pregnancy and infancy, which suggested that oral health care should be paid attention to as early as possible in infants with good nutritional status to prevent the occurrence of dental caries.

There remain some limitations in this study. First, the cross-sectional survey method was used to calculate the eruption age of primary teeth, which may be bias compared with the data obtained from longitudinal studies, as well as the sequence and duration of primary teeth eruption were not considered. Long-term longitudinal studies are difficult to implement, so we only discussed the changes of the eruption age of the primary teeth in different time using the cross-sectional surveys like most population studies. Second, considering the feasibility and the accuracy of information collection in large-scale population surveys, we only investigated the number of erupted primary teeth of infants and young children, but did not record the position of the primary teeth. Third, we only analyzed the trends of primary teeth eruption among infants and young children under 24 months because the primary teeth erupted data was not collected among young children above 24 months in 1995, which may not cover all trends of primary teeth eruption because of around 28–32 months for all the primary teeth erupted.

In conclusion, the primary teeth eruption slightly advanced with social development, and their gender difference and regional difference have always existed, which supplied some data for understanding the secular trend of primary teeth development in anthropology and other related fields, and supplied some evidence for evaluating primary teeth eruption and formulating policies in the oral health care and the early prevention of dental caries in stomatology and pediatrics. In the future, more primary teeth development data from various populations based on the same research methods is necessary to clarify whether there is an advanced trend and their related factors.

## Data Availability

The data that support the findings of this study are available from the corresponding author upon reasonable request.
